# The effect of fluoride iontophoresis on seal ability of self-etch adhesive in human dentin in vitro

**DOI:** 10.1186/s12903-022-02146-w

**Published:** 2022-04-02

**Authors:** Kanittha Kijsamanmith, Parintorn Wallanon, Chanya Pitchayasatit, Poonnapha Kittiratanaviwat

**Affiliations:** 1 Department of Oral Biology, Faculty of Dentistry, Mahidol University, Yothi Road, Bangkok, 10400 Thailand; 2 Mahidol International Dental School, Faculty of Dentistry, Mahidol University, Bangkok, Thailand

**Keywords:** Caries affected dentin, Human dentin, Fluoride iontophoresis, Seal ability, Self-etch adhesive

## Abstract

**Background:**

Fluoride iontophoresis (FI) is a non-invasive method for the transfer of fluoride ions under electrical pressure into dental hard tissue. This study aimed to determine the effect of FI on the seal ability of self-etch adhesive in human dentin using dentin permeability test and scanning electron microscopy (SEM).

**Methods:**

The experiments were divided into 2 series: series 1 was performed on 28 extracted intact third molars and series 2 was performed on 28 extracted carious third molars (ICDAS 4 and 5). In each series, 20 teeth were used for dentin permeability test and 8 teeth were used for SEM study. For dentin permeability test, the specimens were divided into dentin without FI (control) and dentin with FI (experimental) subgroups. Hydraulic conductance (HD) of dentin was measured before and after adhesive treatment, and calculated for the percentage decrease of HD in each subgroup. Two-way ANOVA and Tukey test were used for statistical analysis. SEM study was used to assess the seal ability of self-etch adhesive and penetration of fluoride ions into dentinal tubules.

**Results:**

HD after self-etch adhesive treatment reduced by 57.75 ± 17.99% in intact dentin with FI, 46.60 ± 17.03% in intact dentin without FI, 45.00 ± 15.30% in caries affected dentin without FI, and 37.28 ± 14.72% in caries affected dentin with FI. There was no significant difference in percentage decrease of HD between dentin without FI and dentin with FI (*P* = 0.742); meanwhile, intact dentin with FI had significant greater percentage decrease than caries affected dentin with FI (*P* < 0.05). SEM findings showed FI produced more particle formation and deeper precipitation in intact dentin than those in caries affected dentin.

**Conclusions:**

FI did not affect the seal ability of self-etch adhesive in human dentin when compared to without FI. However, FI could augment the seal ability of the self-etch adhesive in intact dentin better than that in caries affected dentin.

**Supplementary Information:**

The online version contains supplementary material available at 10.1186/s12903-022-02146-w.

## Background

Dental caries is one of common chronic diseases which affects the quality of life all over the world. The treatment of dental caries mainly consists of caries removal and tooth restoration which based on the international caries detection and assessment system (ICDAS) and the international caries classification and management system (ICCMS™) [1, 2]. For deep dentinal caries lesions, the use of selective carious tissue removal has advantage in terms of avoidance of pulp exposure, preservation of dental and pulpal tissues as well as the longevity of the restorations [2, 3]. In the selective carious tissue removal, the carious tissue was removed with hand instrument until the dentin started to become firm and leathery; thereafter, the caries affected dentin was bonded to the adhesive material for prevention of post-operative dentin sensitivity and pulpal inflammation [2, 3]. The dental adhesive was used to provide an immediate dentin seal to protect pulpo-dentin complex, and two step self-etch adhesive gave an excellent seal in vitro [4].

Fluoride has ability to reduce demineralization and enhance remineralization of dental hard tissue; therefore, fluoride is significantly important for caries prevention and treatment [5, 6]. Generally, fluoride toothpaste and other fluoride containing products such as fluoride mouthrinse are accepted to use for anticaries benefits [6]. In addition, sodium fluoride was recommended to treat dentin hypersensitivity by Lukomsky [7]. The topical application of 2% sodium fluoride solution on exposed dentin caused a desensitization effect due to fluoride penetration and precipitation of calcium fluoride within dentinal tubules; however, topical sodium fluoride treatment took times to form calcium fluoride crystals and had no immediate post treatment effect [8, 9]. Fluoride iontophoresis (FI) is a simple, non-invasive method for the transfer of fluoride ions under electrical pressure into dental hard tissue such as enamel, dentin [9, 10]. The application of FI could promote both enamel remineralization and occlusion within dentinal tubules in order to control dental caries and reduce dentin hypersensitivity [11, 12]. Huang and Guo [13] found that 2% sodium FI produced greater size of precipitates formed and deeper fluoride penetration, when compared to fluoride treatment without iontophoresis. Furthermore, 2% sodium FI showed greater long term desensitization effect than HEMA-G and topical fluoride application [9, 14]. In operative procedure of intact dentin, FI did not significantly reduce the bond strength of a self-etch adhesive system [15].

Although, iontophoresis could enhance drug delivery through both intact and caries affected dentin, the drug diffusion rate in caries affected dentin was lower than that in intact dentin [16]. Additionally, the caries affected dentin affects the dental adhesive in terms of lower bond strength and poor quality of the hybrid layer, when compared to intact dentin [17]. Beside the bond strength, durability and dentin sealing ability of adhesive should be evaluated. The capacity of dentin sealing ability is considered for post-operative success in terms of reduction of dentin hypersensitivity and prevention of pulpal inflammation [4]. Several studies of seal ability of adhesives used dentin permeability test to determine the fluid movement across the adhesive layer in dentinal tubules under various hydrostatic pressures [18–20].

Recently, Bertolo et al. [21] found iontophoresis improved dentinal infiltration and reduced nanoleakage of adhesive systems. However, there was no study evaluated the effect of FI on seal ability of adhesive in human dentin in vitro. Therefore, the aim of the present study was to determine the effect of FI on seal ability of self-etch adhesive in human intact and caries affected dentin using dentin permeability test and scanning electron microscopy (SEM).

## Methods

The study was approved by the Institutional Review Board, Faculty of Dentistry/Faculty of Pharmacy, Mahidol University, Thailand (COE.No.MU-DT/PY-IRB 2020/020.1506), and complied with the principles of the Declaration of Helsinki. The experiments were divided into 2 series: series 1 was performed on 28 extracted intact human third molars and series 2 was performed on 28 extracted carious human third molars (ICDAS 4 = 20 teeth, ICDAS 5 = 8 teeth) [1]. In each series, 20 teeth were randomly selected for dentin permeability test and 8 teeth were randomly selected for SEM study. The human third molars were obtained from Oral and Maxillofacial Surgery Clinic, Dental Hospital, Faculty of Dentistry, Mahidol University. All teeth were extracted for dental reasons, such as dental caries and its consequences, eruption problems, periodontal diseases, prophylactic removal, and orthodontic purposes. After extraction, the teeth were immediately kept in 0.1% thymol solution (M Dent, Nakhon Pathom, Thailand), and used within 2 weeks. The teeth with crack, craze line, history of root canal treatment, or crown restoration were excluded.

### Tooth preparation

In series I, each intact human third molar was sectioned transversely 3 mm below and above the cementoenamel junction with a diamond disc under water coolant. In series II, each carious human third molar was sectioned transversely 3 mm below and above the cementoenamel junction with a diamond disc under water coolant. After that, soft carious tissue was removed using spoon excavator according to the principle of selective carious tissue removal, until the dentin became firm and leathery [2, 3]. Thereafter, in both series, coronal pulpal tissue was removed with tweezer, and the pulp cavity was cleaned with distilled water to remove any remnant tissue. Each crown specimen was glued to an acrylic block which connected to the fluid filtration device following the protocol of Kijsamanmith et al. [22].

### Dentin permeability test

**Fig. 1:**
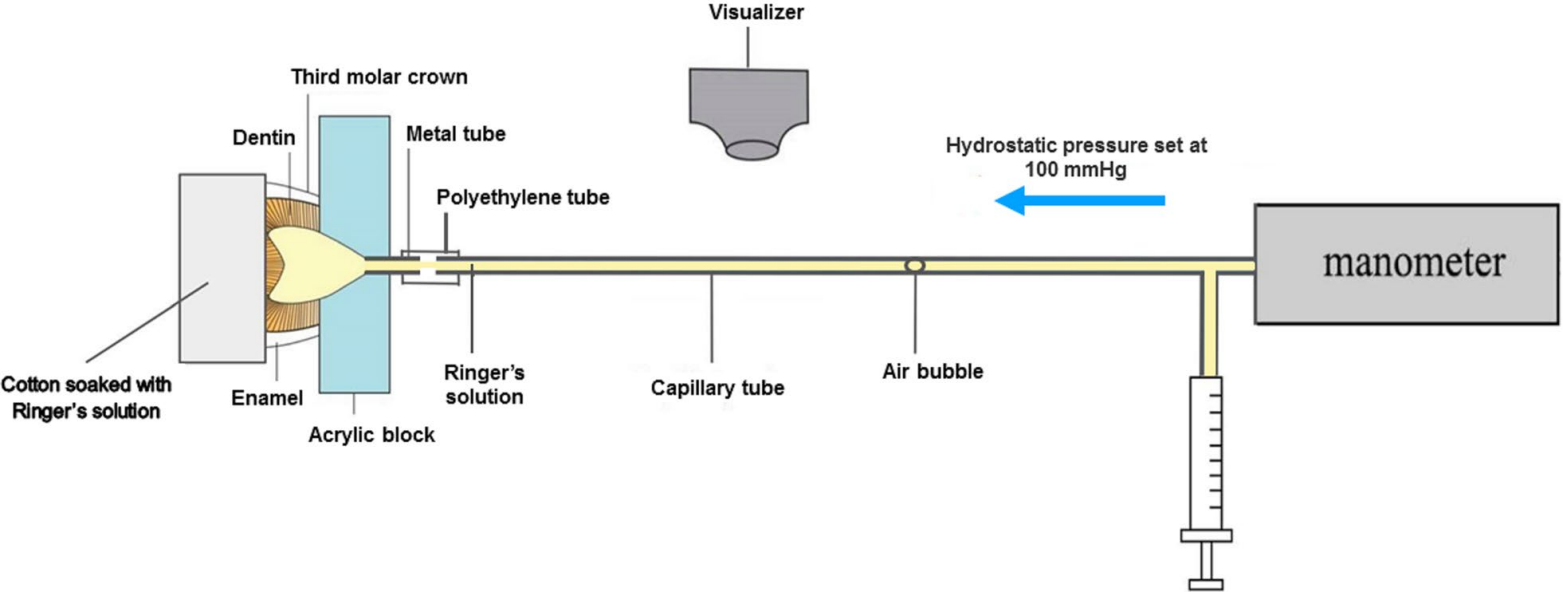
Diagram of the experimental set up for dentin permeability test

Twenty teeth in each series were randomly divided into 2 subgroups; subgroup 1: dentin without FI (control) and subgroup 2: dentin with FI (experimental). After tooth preparation, the exposed occlusal dentin was covered with cotton soaked with Ringer’s solution to prevent dentin dehydration. Baseline hydraulic conductance of dentin with smear layer (before treatment) was measured by observing the movement of a small air bubble which introduced into a capillary using visualizer and the pressure in the fluid filtration system was set at 100 mmHg above atmospheric using a mercury manometer (Fig. 1) [23]. Thereafter, in group of dentin with FI, the occlusal dentin covered with cotton soaked with a neutral 2% sodium fluoride solution (900 ppm fluoride; Duraphat®, USA). Then, the tooth specimen was connected with iontophoresis device (DENTAPHOR™ –II MODEL 6111D, Life-Tech Inc®, USA) for applying cathode electric current with fluoride at 0.5 mA for 5 min [15] under intrapulpal pressure of 11 mmHg [24, 25]. After that, both dentin without and with FI were treated with 2-step self-etch adhesive (Clearfil™ Liner Bond F; Kuraray Noritake Dental Inc®, Japan), and light-cured with a halogen lamp-curing unit (3 M ESPE Elipar ™ 2500, USA) according to the manufacturer’s instructions. The hydraulic conductance of bonded dentin (after treatment) was measured again as described above. Thereafter, hydraulic conductance of dentin before and after treatment were calculated for the percentage decrease of hydraulic conductance in each subgroup.

### SEM study

Eight teeth of each series were prepared and randomly divided into different conditions: dentin with smear layer (n = 2), dentin with self-etch adhesive treatment (n = 2), dentin with FI (n = 2) and dentin with FI + self-etch adhesive treatment (n = 2) for SEM study using a scanning electron microscope (JSM-5410 LV; JEOL, Tokyo, Japan) in both cross sectional views and longitudinal views. Specimens of dentin under different conditions were observed to assess the characteristics and thickness of smear layer, adhesive layer, size and form of fluoride precipitation and depth of fluoride penetration within dentinal tubules.

### Statistical analysis

The HD of dentin before and after treatment and the percentage decrease of HD in each group are represented as mean and standard deviation (SD). The mean percentage decreases of HD were compared using two-way analysis of variance (2-way ANOVA). Where this showed a significant effect, Tukey test was used for pairwise multiple comparisons. *P* values less than 0.05 were considered significant.

## Results

### Hydraulic conductance of dentin

**Fig. 2:**
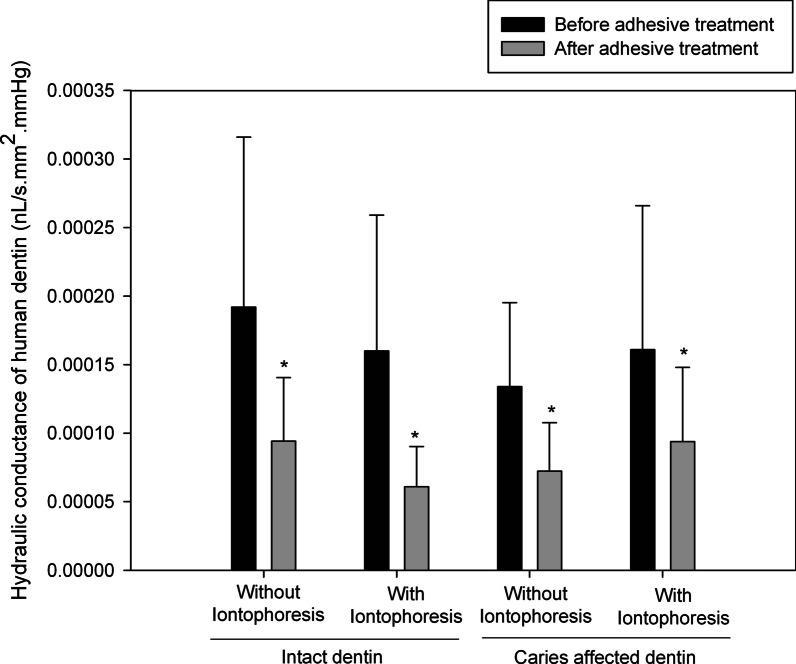
Mean (± 1 SD) hydraulic conductance values of dentin without and with fluoride iontophoresis before (black column) and after (gray column) adhesive treatment. *Statistically significant reductions after adhesive treatment (*P* < 0.05, paired *t*-test)

The means and SDs of HD of both intact and caries affected dentin, without and with FI, and before and after self-etch adhesive treatment are shown in Fig. 2. There was a significant difference between before and after adhesive treatment in each subgroup (*P* < 0.05, paired *t*-test).

**Fig. 3:**
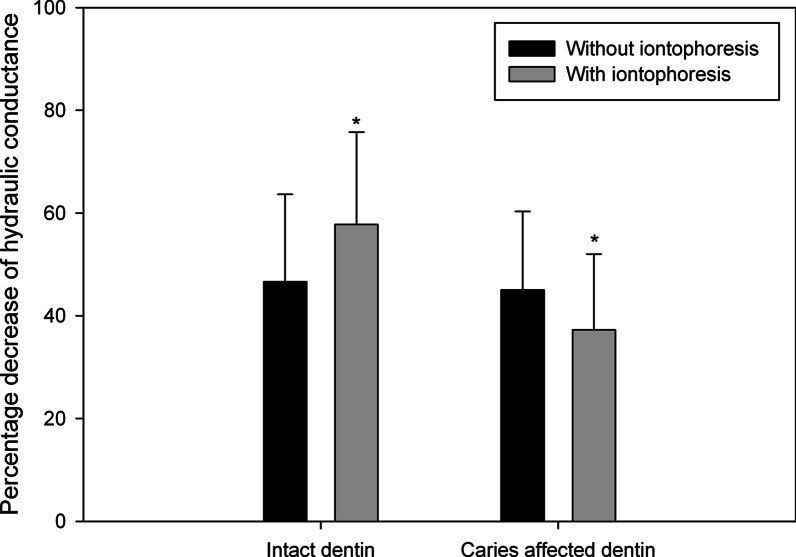
Mean (± 1 SD) percentage decreases of hydraulic conductance of dentin without (black column) and with (gray column) fluoride iontophoresis after self-etch adhesive treatment. * Statistically significant difference between intact dentin with FI and caries affected dentin with FI (*P* < 0.05, 2-way ANOVA and Tukey test)

After self-etch adhesive treatment, the means and SDs of percentage decrease of HD of intact and caries affected dentin without and with FI, are shown in Fig. 3. HD after self-etch adhesive treatment reduced by 57.75 ± 17.99% in intact dentin with FI, 46.60 ± 17.03% in intact dentin without FI, 45.00 ± 15.30% in caries affected dentin without FI, and 37.28 ± 14.72% in caries affected dentin with FI, respectively. There was no significant difference in percentage decrease of HD of dentin between without and with FI (*P* = 0.742); meanwhile, intact dentin with FI had significant greater percentage decrease of HD than caries affected dentin with FI (*P* < 0.05).

### SEM findings

**Fig. 4:**
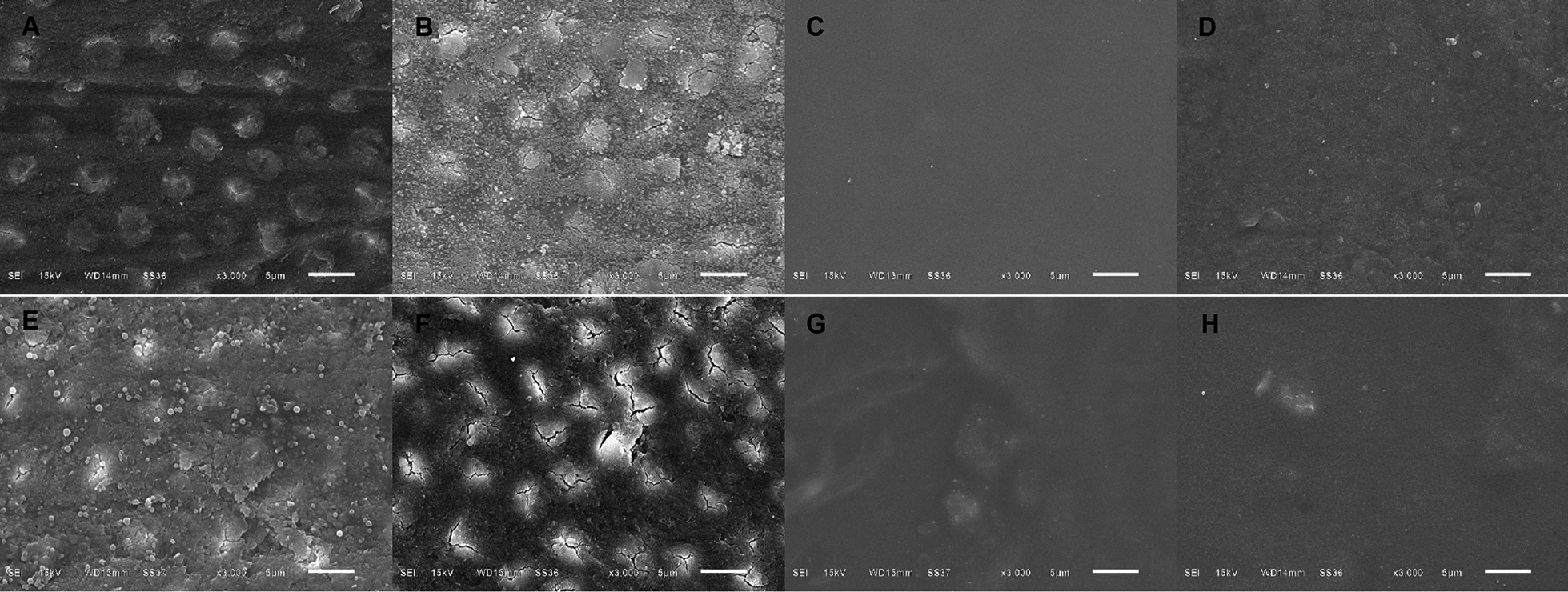
**A**–**H** Scanning electron micrographs of cross sectional views of human dentin (original magnification X3000). **A**–**D** Intact dentin; **E–H** caries affected dentin; **A**, **E** smear layer covered dentin and no treatment; **B**, **F** with fluoride iontophoresis; **C**, **G** with self-etch adhesive treatment; **D**, **H** with fluoride iontophoresis and adhesive treatment

**Fig. 5:**
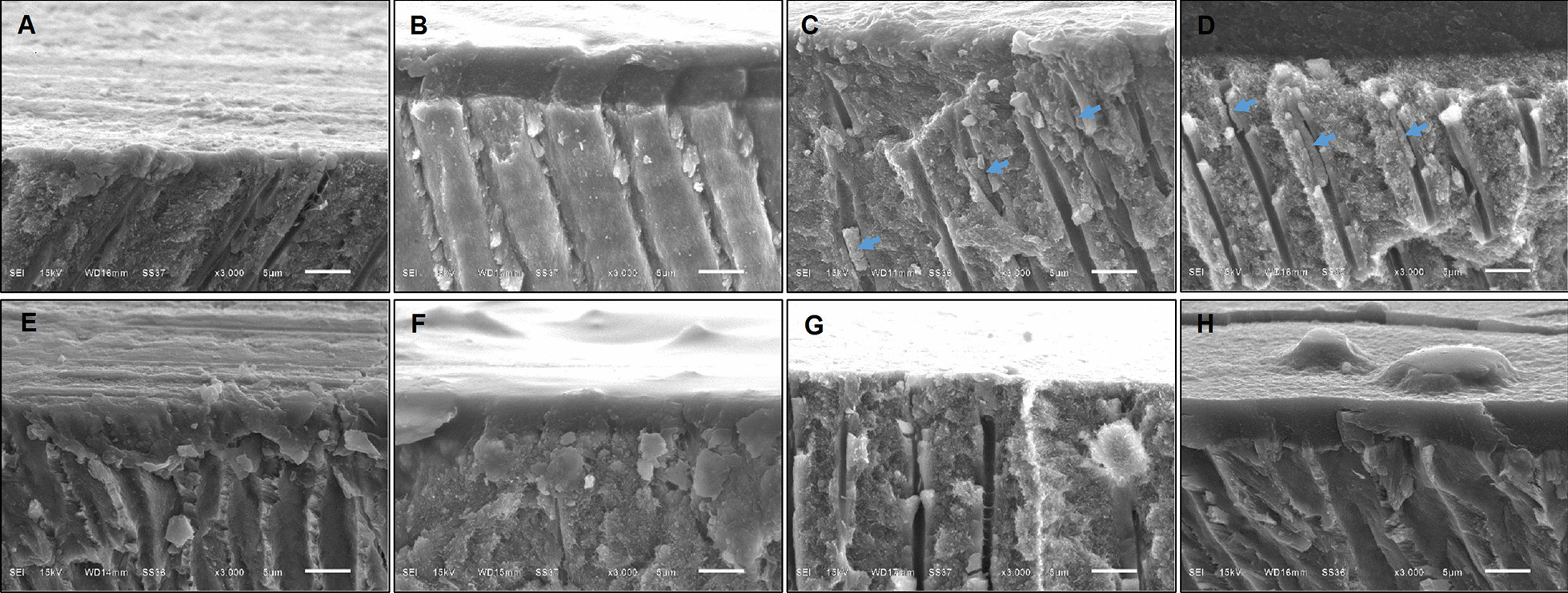
**A**–**H** Scanning electron micrographs of longitudinal views of human dentin (original magnification X3000). **A**–**D** Intact dentin; **E**–**H** caries affected dentin; **A**, **E** smear layer covered dentin and no treatment; **B**, **F** with self-etch adhesive treatment; **C**, **G** with fluoride iontophoresis; **D**, **H** with fluoride iontophoresis and adhesive treatment. Arrow heads showing bundle precipitates formed within dentinal tubules of intact dentin with fluoride iontophoresis

**Fig. 6:**
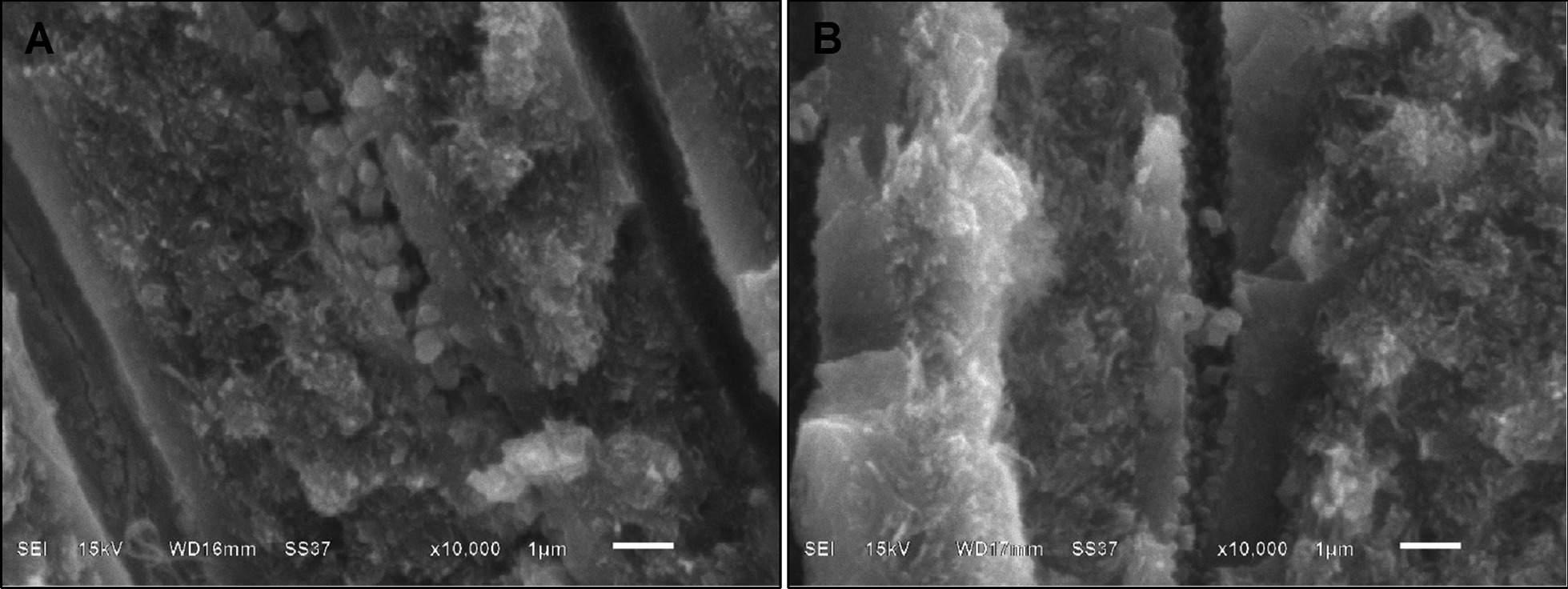
**A**, **B** Scanning electron micrographs of longitudinal views of human dentin (original magnification X10000), showing granular precipitates in dentinal tubules. **A** Intact dentin at the depth of 300 µm; **B** caries affected dentin at the depth of 60 µm

The smear layer of intact dentin was smooth and compact with thickness of 2 µm (Figs. 4A, 5A); meanwhile, caries affected dentin had a thicker smear layer of 5 µm with irregular pattern of debris (Figs. 4E, 5E). After FI, at dentin surface, intact dentin had larger numbers of scattering fluoride granules compared to caries affected dentin (Fig. 4B, F). In addition, within the dentinal tubules, intact dentin represented greater fluoride penetration than caries affected dentin. Bundle precipitations with the size of 4–10 µm were found in intact dentin at the depth of 8–30 µm (Fig. 5C, D), but it was not found in caries affected dentin (Fig. 5G, H). Larger quantities of granular precipitation were also found in dentinal tubules of intact dentin with larger in size, at the deeper level of 300 µm (Fig. 6A); meanwhile, only small granular precipitations were found at the depth of 60 µm in dentinal tubules of caries affected dentin (Fig. 6B). Nevertheless, after self-etch adhesive treatment in both dentin without and with FI, the adhesive layer looked similar (Fig. 4C, D, G, H) with the thickness of 5–6 µm (Fig. 5B, D, F, H, Additional file 1: Fig. S1).

## Discussion

This study demonstrated that after self-etch adhesive treatment, HD reduced by 57.75 ± 17.99% in intact dentin with FI, 46.60 ± 17.03% in intact dentin without FI, 45.00 ± 15.30% in caries affected dentin without FI, and 37.28 ± 14.72% in caries affected dentin with FI. This result suggested that the best dentin sealing ability found in intact dentin with FI, followed by intact dentin without FI, caries affected dentin without FI, and caries affected dentin with FI, respectively. Although 2-step self-etch adhesive could not completely seal the dentin (100%), it lowered the HD and provided better sealing of dentin, when compared to dentin with smear layer (before adhesive treatment). Consistent with the SEM micrographs of our study, the presence of the bonding layer of self-etch adhesive covered all the orifices of dentinal tubules, and represented better tight seal than smear layer covered dentin. This immediate dentin seal of self-etch adhesive helps in prevention of bacteria leakage and pulpal inflammation [4]. Sealing the dentinal tubules with dental adhesive was also suggested to produce long lasting blockage of dentin hypersensitivity [26], due to its possessing a high sealing ability for dentin surface and significantly reducing dentin permeability [27]. In randomized clinical trials, dental adhesive had ability in decreasing dentin hypersensitivity following periodontal surgeries [28, 29].

The present study also demonstrated that there was no significant difference in percentage decrease of HD of bonded dentin between without and with FI; hence, FI did not affect the seal ability of self-etch adhesive in human dentin when compared to without FI. In agreement with the study of Chen et al. [15], FI did not significantly reduce the bond strength of a self-etch adhesive system. In this study, Clearfil Liner Bond F was used due to its fluoride-releasing adhesive at the bonded interface. Clearfil Liner Bond F creates the thicker super dentin layer than Clearfil SE Bond, leading to strengthening teeth and preventing dental caries [30, 31]. Fluoride-releasing two-step self-etch adhesive is easy to use, gives chemical bond to dentin substrate, maintains hydroxyapatite of dentin, and keep its bonding durability to tooth structure [32].

However, our study found that FI produced greater percentage decrease of HD or better sealing ability, deeper fluoride penetration and more particle precipitation within dentinal tubules of intact dentin than those of caries affected dentin. Thus, FI could augment the seal ability of the self-etch adhesive in intact dentin better than that in caries affected dentin. This may be due to the different in characteristics and properties of intact and caries affected dentin [17]. Furthermore, the smear layer formed on caries-affected dentin is different in morphological and chemical structures from the smear layer formed on intact dentin [17]. In the SEM micrographs of our study, the smear layer of caries affected dentin, was thicker, more irregular and enriched with organic components when compared with the smear layer of intact dentin. Hence, these differences might affect the ability of FI in intact and caries affected dentin.

Currently, the application of adhesive systems with an electric current transmission device was developed to improve the impregnation of the adhesive into intact dentin [21]. The electric currents increased the microtensile bond strengths in 24 h storage, and reduced the adhesive system’s nanoleakage after 1-year storage in vitro [21]. Meanwhile, cathode FI of the present study is the use of 2% sodium fluoride combined with an iontophoresis device, a non-invasive method, for the transfer of fluoride ions under electrical pressure into dental hard tissue. This is the first study to demonstrate that FI had no adverse effect on the seal ability of self-etch adhesive in both intact and caries affected dentin, and FI could augment the seal ability of the self-etch adhesive in intact dentin better than that in caries affected dentin. In addition, it was well documented that fluoride is significantly beneficial in the dental field for caries prevention and treatment, including the reduction of tooth demineralization, enhancement of tooth remineralization, interference with dental plaque formation and inhibition of microbial activity [5, 6, 33]. 2% sodium fluoride also helps in reducing dentin hypersensitivity, and its effectiveness increases when treated with cathode FI [9]. Consistent with the study of Huang and Guo [13], dentin treated with 5-min FI achieved larger particle size and deeper fluoride penetration in dentinal tubules than dentin treated with 5-min topical fluoride application. Thus, FI is preferred technique to provide long lasting desensitizing effect [9, 14], and to improve remineralization effect [10, 34]. During FI, the fluoride ions in solution come into contact with the cathode and move towards to dental hard tissue (the anode); meanwhile, the electric currents facilitate the release of calcium and phosphate ions from hydroxyapatite to form calcium fluoride and fluoroapatite crystals [35]. These are reasons why FI caused a lot of particle precipitations in dentinal tubules at various depths in human dentin. Thus, FI not only provides multi-benefit effects for dentin but also can be used in combination with self-etch adhesive application.

With limitation in this study, the seal ability of bonded dentin was measured only immediately after self-etch adhesive treatment; however, further studies should investigate the durability of sealing ability of bonded dentin. Randomized clinical trials are also needed to support the results of the present study.

## Conclusions

FI did not affect the seal ability of self-etch adhesive in human dentin when compared to without FI. However, FI produced greater percentage decrease of HD, deeper fluoride penetration and more particle precipitation within dentinal tubules of intact dentin than those of caries affected dentin. Thus, FI could augment the seal ability of the self-etch adhesive in intact dentin better than that in caries affected dentin.

## Supplementary Information

**Additional file 1: Fig. S1**. Scanning electron micrographs of intact dentin with fluoride iontophoresis and self-etch adhesive treatment.

## Data Availability

The datasets used and/or analyzed during the current study are available from the corresponding author on reasonable request.

## Abbreviations

FI: Fluoride iontophoresis
SEM: Scanning electron microscopy
HD: Hydraulic conductance
ICDAS: International caries detection and assessment system
