# Supplementary material for: The effect of fluoride iontophoresis on seal ability of self-etch adhesive in human dentin in vitro
Source: BMC Oral Health. 2022 Apr 2;22:109. doi: 10.1186/s12903-022-02146-w (PMC8976950; doi:10.1186/s12903-022-02146-w)
Supplement: Supplementary file 1 — Additional file 1: Fig. S1. Scanning electron micrographs of intact dentin with fluoride iontophoresis and self-etch adhesive treatment. [file 12903_2022_2146_MOESM1_ESM.pdf]

A.
B.
C.

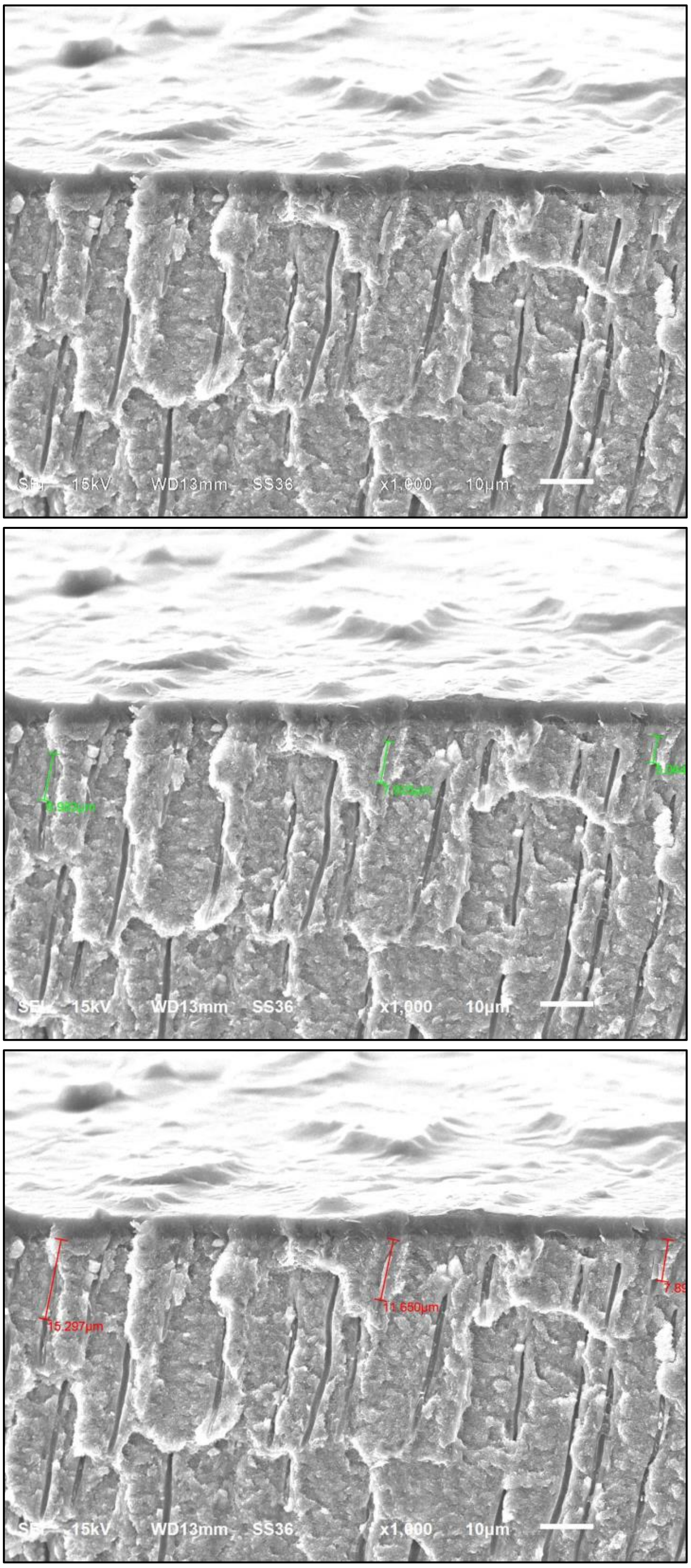

Figure S1 (A–C) Scanning electron micrographs of longitudinal views of intact dentin with fluoride iontophoresis and self-etch adhesive treatment (original magnification X1000), showing bundle precipitates with the size of 5-10 µm in dentinal tubules at the depth of 8-16 µm.
